# A 5-day antibiotic stewardship target for uncomplicated community-acquired pneumonia

**DOI:** 10.1017/ash.2026.10770

**Published:** 2026-07-09

**Authors:** Shuchi Amin, Andrea Call, Jill Bellows, Christopher Noel, Joseph Miles, Andrew LaPoint, Aaron Shaykevich, Ivayla I. Geneva

**Affiliations:** 1 Crouse Hospital, USA; 2 State University of New York Upstate Medical University, USA

## Abstract

**Introduction:**

Antibiotic overuse in community-acquired pneumonia (CAP) persists despite guidelines recommending 5-day treatment for uncomplicated cases. Our pilot quality improvement study aimed to address this problem via an antibiotic stewardship initiative.

**Methods:**

Adults admitted to a tertiary care center with uncomplicated CAP were identified using database query and manual chart review, with exclusions for intensive care needs, lack of clinical improvement, or concurrent infections. A 3-month baseline period was compared to a 3-month post-intervention period after provider-targeted interventions were implemented—in-person and email-based provider education on eligibility for the 5-day antibiotic course and pharmacist-driven outreach on day 3 of treatment to encourage guideline-based prescribing. Mean antibiotic duration was compared using Mann–Whitney test. The primary goal was to decrease antibiotic overuse by 25%.

**Results:**

Of 647 baseline patients, 222 met criteria with a mean antibiotic duration of 6.75 ± 1.75 days. After the interventions, 170 of 570 patients met criteria with a reduced antibiotic duration of 6.24 ± 1.47 days (*P* = .001). The antibiotic overuse decreased by 28.7%. Prolonged courses were primarily due to habitual use of traditional longer regimens, unaccounted inpatient or emergency department doses at discharge, and discrepancies between documentation and medication orders.

**Conclusions:**

Our pilot study showed that a provider-focused pharmacist-driven strategy can reduce unnecessary antibiotic use in uncomplicated CAP. The intervention encouraged shorter, guideline-driven courses and reduced antibiotic overuse by 28.7%, meeting our primary goal of 25% reduction. Nevertheless, there are opportunities for further improvement in guideline adherence through enhanced discharge prescription stewardship, documentation accuracy checks, and continued provider education.

## Introduction

Antibiotic stewardship is a cornerstone of modern healthcare as antibiotic overuse continues to fuel antimicrobial resistance, drug-related complications, and rising healthcare costs.^1,2^ Despite widespread awareness and published guidelines, prolonged antibiotic courses remain common in both hospital and outpatient settings.^3^ A major challenge to adherence is translating clinical guideline recommendations into consistent bedside practice, particularly when factoring in clinicians’ behavior in the face of diagnostic uncertainty, clinicians’ habits, and patient complexity, all of which may influence prescribing decisions.^4–6^ Community-acquired pneumonia (CAP) is among the most common infections requiring hospitalization and is a leading driver of antibiotic use in the United States.^7^ Unnecessary extension of treatment beyond guideline-recommended durations contributes significantly to overall antibiotic exposure.^8^ The current American Thoracic Society and Infectious Diseases Society of America (ATS-IDSA) guidelines advise that a 5-day antibiotic treatment would be adequate for uncomplicated CAP cases that show clinical improvement.^9^ Although evidence supporting this short 5-day course is robust, provider adherence to the guideline-driven recommendations remains low.^10^ Our quality improvement (QI) study addresses this problem by implementing a provider-targeted stewardship strategy focused on the management of uncomplicated CAP.

## Methods

**Figure 1.**
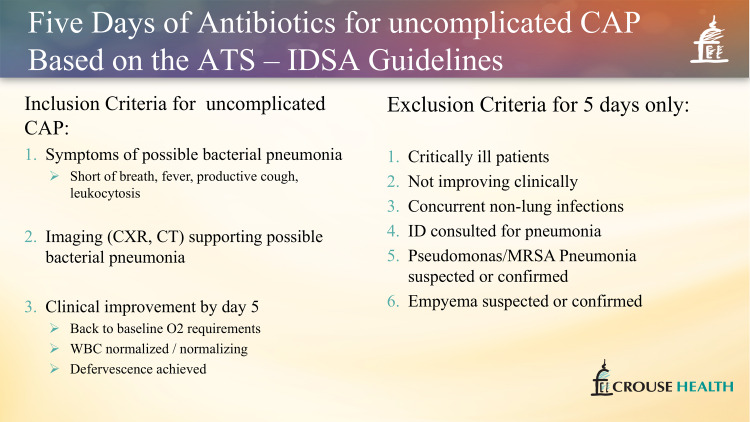
Education slide on the diagnosis and management of community-acquired pneumonia. Abbreviations: CAP: community-acquired pneumonia, ATS, American Thoracic Society; IDSA, Infectious Diseases Society of America; CXR, chest X-ray; CT, computed tomography; ID, infectious diseases; MRSA, methicillin-resistant *Staphylococcus aureus*.

This is a pilot QI study of patients admitted to a tertiary medical center (Crouse Hospital in Syracuse, NY) from December 2024 to May 2025. The following provider-targeted QI intervention took place at the end of February 2025 to encourage guideline-based antibiotic duration for the treatment of uncomplicated CAP. We targeted all hospital providers, including physicians and mid-level providers. The intervention constituted an in-person and email-based education regarding the inclusion and exclusion criteria for the recommended short 5-day antibiotic course. A slide describing the criteria (Figure 1) was shared via email with all prescribers in the hospital. Additionally, the slide was presented via ZOOM to the hospitalists’ group (physicians and mid-level providers) during their monthly meeting. The slide was then presented again on monthly basis during the hospitalist group’s monthly meetings. At our hospital, the hospitalist group manages ∼65% of all patients in the hospital. The attendance at the monthly hospitalist meeting is on average 80% of the hospitalist physicians and mid-level providers. Concurrently, starting in March 2025, we implemented pharmacist-driven antibiotic stewardship, where all non-intensive care unit (ICU)-based prescribers managing adult patients with pneumonia were contacted during the week (Monday through Friday) in-person or over phone on day 3 of antibiotic treatment to encourage a 5-day courses for their patients if they met criteria for uncomplicated CAP as defined in the 2025 ATS-IDSA guidelines.^9^ Prescribers were contacted by the pharmacist only once during the hospitalization regarding the treatment of CAP, and we did not perform stewardship oversight regarding antibiotics prescribed for outpatient use on the day of hospital discharge. Patients for the inclusion in our detailed chart review were identified as follows. We performed a database query to identify patients who received antibiotics for pneumonia. Using manual chart review, we identified those who met the following criteria for uncomplicated CAP. The inclusion criteria were: adults (age ≥ 18 yr), presenting symptoms and signs consistent with pneumonia such as the presence of cough, fever (recorded body temperature of ≥ 38.0), dyspnea (the patient reporting shortness of breath), hypoxia (SpO2 < 89% on room air; for people using oxygen supplementation at base, SpO2 < 89% while using their baseline oxygen), chest X-ray and/or CT scan showing consolidation, ground-glass opacities, or infiltrates. The exclusion criteria were: need for intubation, management in the ICU, additional infections, confirmed or suspected MSSA or MRSA or Pseudomonas pneumonia, confirmed or suspected empyema, or by day 5 of antibiotic treatment lack of clinical improvement such as continuation or worsening of symptoms, persistent oxygen requirement above baseline, or non-resolving leukocytosis. We ensured reproducibility in the identification of cases by screening all patients twice—first by JB and second by SA, with approximately 90% agreement, where the disagreed-upon cases were arbitrated by the review of a third researcher, IIG. For patients meeting the inclusion and exclusion criteria, we compared the patient characteristics of the baseline versus postintervention cohorts using age, sex, CURB-65, microbiology results for respiratory tract infections, length of stay (LOS) normalized to the overall LOS for hospitalized patients during the same time periods (calculated using monthly LOS averages) and with outliers (LOS >20 d) removed, and disposition (home vs rehab), mortality, 30-day all-cause readmission rates and 30-day readmission rate for recurrent pneumonia. We statistically compared the above-listed variables using the following statistical tests where appropriate: χ^2^, Mann–Whitney test, and Fisher exact. We noted the baseline duration of antibiotic courses over 3 months (December 2024 to February 2025) and the postintervention antibiotic duration over 3 months (March 2025 to May 2025). In our antibiotic duration calculations, we included antibiotics administered in the emergency room, during the hospitalization period, and the additional antibiotics prescribed on discharge, if any. We used calendar days. For example, if a patient was admitted at 8 AM and received the first dose of antibiotic at 8 AM was handled the same way as a patient admitted at 8 PM and having received their first antibiotic dose at 8 PM. A total antibiotic course was calculated by summing the 24-hour periods during which patients received antibiotics for pneumonia while in the hospital and the 24-hour periods of prescribed outpatient antibiotics, if any were prescribed on the day of discharge from the hospital. The Mann–Whitney test was used to evaluate for statistical significance when comparing the before-and-after intervention antibiotic use duration. The primary stewardship objective was to achieve a 25% decrease in the antibiotic overuse, where overuse is calculated as the number of treatment days above the recommended 5-day course.

## Results

### Patient demographics and hospitalization characteristics before and after the quality improvement intervention

There were a total of 1,217 patients who received antibiotics for pneumonia during the 6-month study period. Approximately 70% of these were contacted by the antibiotic stewardship pharmacist during their Monday–Friday coverage. Of those that were contacted, approximately 80% had agreed to the stewardship suggestions regarding adherence to the ATS-IDSA guidelines. From the 647 patients treated with antibiotics for pneumonia before our QI intervention, 222 met the inclusion and exclusion criteria for uncomplicated CAP and appropriateness for the 5-day antibiotic course. During the 3 months following the intervention, there were 570 patients treated for pneumonia, from whom 170 met our criteria. Table 1 provides the patient demographics—age and sex, as well as additional patient characteristics for the pre- and postintervention cohorts—CURB65 score, viral panel positivity (test was for COVID-19, Influenza A, B, and RSV), nares PCR screen for MSSA and MRSA, sputum culture positivity, the normalized LOS, disposition (to home vs to rehab), 30-day all-cause readmission rates, and 30-day readmission rates specifically for the recurrence of pneumonia. Mortality for both groups was at zero. Except for the higher rate of viral panel positivity in the preintervention cohort, all other variables showed no statistical difference between the 2 patient cohorts. Regarding the LOS, the preintervention cohort had 4 outliers with LOS>20, and the postintervention cohort had no outliers. Before removing the outliers and normalizing for the overall decrease in LOS for all hospitalized patients during the respective study periods, the LOS appeared to have decreased following the intervention. The difference disappeared after removal of the outliers and normalization.

**Table 1.** Patient characteristics and statistical comparison of the pre and postintervention cohorts

Variable	Preintervention cohort	Postintervention cohort	*P*-value	Statistics details
Cohort size (# individual patients)	222	170		
Sex			0.545	χ²(1, N = 392) = 0.37, Phi = 0.03
Male	86 (38.7%)	71 (41.8%)		
Female	136 (61.3%)	99 (58.2%)		
Age				
Mean (SD)	72.03 (15.60)	70.85 (16.71)	0.583	Mann–Whitney test = 18,260, *Z* = −0.548
Median (range)	75 (25–105)	73 (16–100)		
CURB65				
Mean (SD)	0.91 (0.87)	0.84 (0.81)	0.505	Mann–Whitney test = 18,176, *Z* = −0.667
Median (range)	1 (0–4)	1 (0–3)		
0	82 (36.9%)	67 (39.4%)	0.425	Fisher exact = 1.22, Cramer’s *V* = 0.057
1	89 (40.1%)	68 (40%)		
2	41 (18.5%)	30 (17.6%)		
3	9 (4.1%)	5 (2.9%)		
4	1 (0.5%)	0 (0%)		
Viral panel			<0.001	χ²(1, N = 392) = 22.58, Phi = −0.24
Negative	154 (69.4%)	152 (89.4%)		
Positive	68 (30.6%)	18 (10.6%)		
Sputum culture			0.788	χ²(1, N = 392) = 0.072, Phi = 0.014
Negative	220 (99.1%)	168 (98.8%)		
Positive	2 (0.9%)	2 (1.2%)		
MRSA/MSSA			0.285	χ²(1, N = 392) = 1.141, Phi = 0.054
Negative	214 (96.4%)	160 (94.1%)		
Positive	8 (3.6%)	10 (5.9%)		
LOS, with outliers, not normalized			0.008	Mann–Whitney test = 15,939, *Z* = −2.656
Mean (SD)	6.19 (5.49)	4.85 (3.09)		
Median (range)	5 (0–54)	4 (1–19)		
LOS, outliers (LOS > 20 d) removed, not normalized			0.017	Mann–Whitney test = 15,939, *Z* = −2.382
Mean (SD)	5.65 (3.46), N = 218	4.85 (3.09), N = 170		
Median (range)	5 (0–18)	4 (1–19)		
LOS, outliers removed, normalized to all hospitalized patients in the time period			0.302	Mann–Whitney test = 17,398, *Z* = −1.033
Mean (SD)	1.24 (0.76), N = 218	1.12 (0.72), N = 170		
Median (range)	1.10 (0–3.95)	0.94 (0.22–4.48)		
Disposition			0.463	χ²(1, N = 392) = 0.539, Phi = 0.037
Rehab	54 (24.3%)	36 (21.2%)		
Home	168 (75.7%)	134 (78.8%)		
Mortality	0	0	NA	NA
30-d all-cause readmission			0.663	χ²(1, N = 392) = 0.190, Phi = 0.022
No	194 (87.4%)	146 (85.9%)		
Yes	28 (12.6%)	24 (14.1%)		
30-d readmission for pneumonia			0.274	χ²(1, N = 52) = 1.20, Phi = −1.52
No	17 (60.7%)	18 (75%)		
Yes	11 (39.3%)	6 (25%)		
Total antibiotics days			0.001	Mann–Whitney test = 15,349, *Z* = −3.265
Mean (SD)	6.75 (1.75)	6.24 (1.47)		
Median (range)	7 (2–14)	6 (3–12)		

χ^2^, χ^2^ test; SD, standard deviation; MRSA/MSSA, methicillin-resistant *Staphylococcus aureus*/methicillin-susceptible *Staphylococcus aureus*; LOS, length of stay; NA, not applicable.

### Decrease in antibiotic utilization following the quality improvement intervention

**Figure 2.**
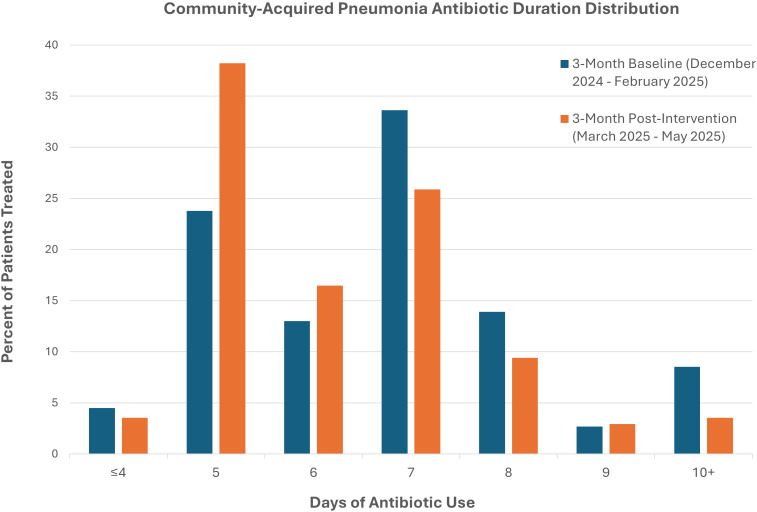
Antibiotic duration for hospitalized patients with uncomplicated community-acquired pneumonia (CAP). Data comparison is shown between a 3-month baseline antibiotic duration from December 2024 to February 2025 (blue) and a 3-month postintervention period from March to May 2025 (red).

**Figure 3.**
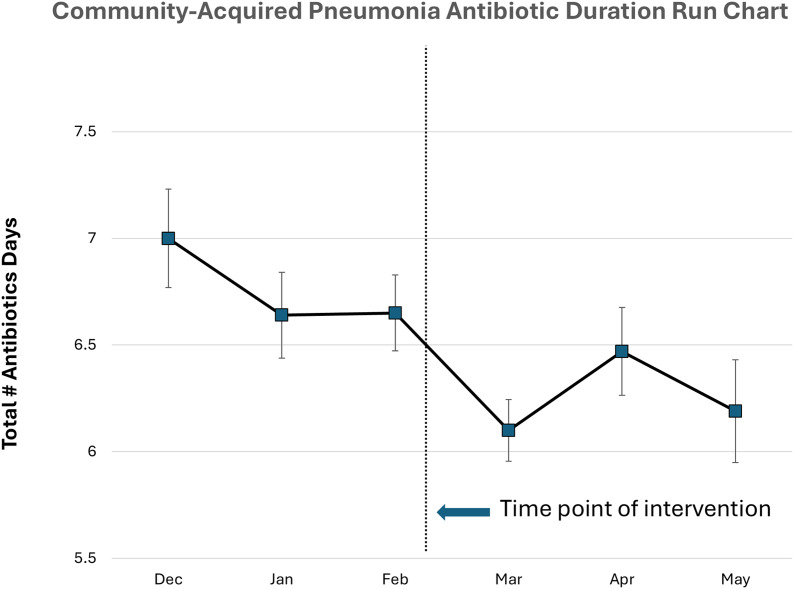
Run chart of antibiotic course duration for community-acquired pneumonia during a 6-month period.

The baseline antibiotic duration was 6.75 ± 1.75 days (standard deviation), and the postintervention antibiotic duration was 6.24 ± 1.47 (Table 1). The Mann–Whitney test yielded a statistically significant result with *P* = .001. Comparing the antibiotic overuse (beyond the guideline-recommended 5 d), the overuse before the intervention was on average 1.74 days, and after the intervention, it decreased to 1.24 days. Therefore, we had achieved a 0.5-day decrease in overuse of antibiotics following the QI intervention, which corresponds to a 28.7% decrease in antibiotic overuse. Figure 2 features a more detailed data comparison between the 3-month baseline and 3-month postintervention period. It provides the percentage of patients treated with antibiotics for a specific number of days. The bar graphs illustrate how the most commonly prescribed antibiotic duration in the postintervention period is 5 days, while during the baseline period, the most common antibiotic duration was 7 days. Figure 3 provides a run chart to allow for visualization of the average antibiotic courses on a per-month basis before and after the QI intervention.

A closer review of prescribing patterns in the postintervention period revealed several drivers of prolonged antibiotic use. In 79.8% of antibiotic courses longer than 5 days, prescribers intentionally defaulted to traditional 7- or 10-day courses, based on their documentation in progress notes and discharge summaries. In 15.2% of cases, even though the providers documented their intention to use a 5-day course, the patients were prescribed an additional 6^th^ day of antibiotics at discharge because providers did not account for inpatient doses already administered on the day of discharge. This was also particularly common when patients received antibiotics in the emergency department, which were often overlooked when calculating total course length. Lastly, 10.1% of cases showed discrepancies between the electronic medical record (EMR) documentation and active medication orders that led to unintended extensions of therapy. In these cases, the providers had documented in their notes that antibiotics had been discontinued, but the orders were not updated in the EMR, resulting in additional antibiotic doses being administered. If the latter 2 issues of unaccounted antibiotics on day of discharge and EMR discrepancies were eliminated, then antibiotic duration after our QI intervention would have been lower with an average of 6.10 ± 1.45 days.

## Discussion

Our pilot study demonstrates that provider-targeted stewardship interventions can reduce unnecessary antibiotic use in patients with uncomplicated CAP that show clinical improvement. Per the guidelines,^9^ these patients can be managed with only a 5-day antibiotic course. Following the intervention, there was a 28.7% reduction in antibiotic overuse (from 6.75 ± 1.75 d to 6.24 ± 1.47 d), which was better than our QI goal of 25% reduction in the overuse. This brought adherence a little closer to the ATS-IDSA guidelines. The decrease in antibiotic course did not appear to have any adverse effect as we found no change in the normalized LOS, discharge disposition (to home vs to facility), all-cause 30-day readmission, and 30-day readmission for pneumonia. This is important because undertreatment of infections is the number one concern when considering shortened treatment. Although the decrease may appear modest in absolute terms, our results highlight the feasibility of implementing a practical intervention that engages providers in real-time and provides guidance for their prescribing decisions. Further, we emphasize the clinical significance in terms of aggregate antibiotic days avoided, that is, the total antibiotics days saved across the postintervention cohort was calculated to be 87 days.

Our finding was strengthened by the fact that based on demographics and hospitalization-related patient characteristics, there was no significant difference between the pre- and postintervention cohorts. The only exception to this was the higher prevalence of viral infections among the preintervention patients, which was easily explained by the fact that the preintervention period spanned the winter influenza season while the postintervention cohort was hospitalized during spring, when viral infections were less common in the general population. Further, one would have expected that the presence of a confirmed viral infection would tend to convince medical providers that the virus is the etiology of patient’s symptoms rather than a bacterial pneumonia, and as such, this could only have led to less antibiotic prescribing. Therefore, it stands to reason that the higher prevalence of viral infections in the preintervention group may have blunted the effect of our antibiotic stewardship intervention.

Our finding that in 79.8% of antibiotic courses longer than 5 days, prescribers intentionally defaulted to traditional 7- or 10-day courses, reflecting the vast majority of prolonged antibiotic courses were still due to limited awareness that 5-day durations are now the guideline recommendation duration for uncomplicated CAP that demonstrates clinical improvement. This fact calls for more education targeted at providers, which we are in the process of implementing. Further, we noted that in 15.2% of the cases, the overuse was due to miscalculation of the antibiotic’s duration, and in another 10.1% of the cases, the overuse was due to discrepancies between the provider’s documented intention and the actual antibiotics ordered via the EMR. We plan to address these 2 problems through education of providers and strengthening of the pharmacy-driven antibiotic use oversight. EMR-based interventions are also being considered, where the creation of EMR safeguards for the reconciliation of medication orders and provider documentation would be most beneficial as well as feasible in the current era of artificial intelligence.

Our results are consistent with several other studies targeting CAP for antibiotic stewardship efforts. Foolad *et al*. utilized a similar multifaceted intervention in a multicenter approach, with provider-targeted education and pharmacist-led inpatient feedback for patients who met CAP inclusion criteria over a 6-month period.^11^ With this intervention, they saw a decrease in duration of therapy from 9 to 6 days (*P* < .0001) with 42% adherence to IDSA-recommended guidelines. They too identified factors of clinician hesitancy and discrepancies with EMR as further sources for optimization of antibiotic duration. On a smaller scale, Avdic *et al.* implemented an intervention with education and real-time pharmacy feedback over a 3-month period in a tertiary-care center.^12^ These efforts were successful and showed a decrease in antibiotic duration from 10 to 7 days (*P* < .0001). Compared to the above-cited studies, ours may seem a small step (of only 0.5 d) in the treatment course reduction, but we point out that our study was targeting the absolute minimum of treatment duration (5 d) and our starting point (the baseline duration of 6.75 d) was not too far from the target.

Limitations of our QI study include the following. Due to lack of staffing, the pharmacist-driven antibiotic stewardship was performed only during the week (Monday through Friday), thus allowing for the overlook of ∼25% of the cases. Also, we could not keep track of whether providers who have initially agreed to the suggested 5-day antibiotic course but in the end overprescribed. Further, we had no control over outpatient antibiotic prescribing on the day of discharge from the hospital, thus limiting the impact of the QI intervention. Next, using calendar days rather than 24-hour periods for the calculation of the antibiotic course duration certainly could introduce a bias toward overestimating the duration; however, it is expected that this overestimation will occur both in the pre- and postintervention period and therefore will not affect the change in antibiotic duration following the QI intervention. Finally, pharmacist outreach to providers is an ongoing long-term initiative at our hospital regarding the treatment of CAP. We envision to strengthen the stewardship process via automated stop dates and prescription monitoring on the day of discharge once we transition our electronic medical records to the versatile EPIC platform later this year.

## Conclusions

Our pilot quality improvement project demonstrated that targeted stewardship strategies can achieve reduction in the antibiotic overuse for patients hospitalized with uncomplicated CAP. Addressing the remaining barriers through a combination of additional provider education, multifaceted pharmacist-driven stewardship, and EMR-based oversight has the potential to further align prescribing with guideline-based best practices and thus minimize unnecessary antibiotic exposure.

## Data Availability

The original data is available upon request.
